# Assessing climate change and health provisions among staff in higher education institutions: A preliminary investigation

**DOI:** 10.1371/journal.pone.0304019

**Published:** 2024-05-21

**Authors:** Walter Leal Filho, Maria Alzira Pimenta Dinis, Amanda Lange Salvia, Javier Sierra, Helena Vasconcelos, Claire Henderson-Wilson, Samo Diatta, T. V. Lakshmi Kumar, Maria Gabriela Meirelles, Fernanda Carvalho

**Affiliations:** 1 European School of Sustainability Science and Research (ESSSR), Hamburg University of Applied Sciences, Research and Transfer Centre “Sustainable Development and Climate Change Management”, Interdisciplinary Expert Centre for Climate Change and Health (IECCCH), Hamburg, Germany; 2 Department of Natural Sciences, Manchester Metropolitan University, Manchester, United Kingdom; 3 Fernando Pessoa Research, Innovation and Development Institute (FP-I3ID), University Fernando Pessoa (UFP), Porto, Portugal; 4 Marine and Environmental Sciences Centre (MARE), University of Coimbra, Coimbra, Portugal; 5 European School of Sustainability Science and Research (ESSSR), Hamburg University of Applied Sciences, Hamburg, Germany; 6 Department of Applied Economics, Faculty of Law, Paseo Tomas y Valiente, Research Center on Global Governance, Educational Research Institute, University of Salamanca, Salamanca, Spain; 7 European School of Sustainability Science and Research; Hamburg University of Applied Sciences, Hamburg, Germany; 8 Faculty of Sciences and Technology, University of the Azores, Ponta Delgada, Azores, Portugal; 9 OKEANOS—R&D Centre, University of the Azores, Horta, Portugal; 10 Health Nature Sustainability Research Group, School of Health and Social Development, Deakin University, Geelong, Australia; 11 Laboratoire d’Océanographie des Sciences de l’Environnement et du Climat, Departement de Physique, Université Assane Seck de Ziguinchor, Ziguinchor, Senegal; 12 Centre for Atmospheric Sciences and Climate Studies, SRM Institute of Science and Technology, Chennai, India; 13 School of Environmental Sciences, Jawaharlal Nehru University, New Delhi, India; 14 Faculty of Science and Technology (FCT), University of the Azores, Ponta Delgada, Azores, Portugal; 15 Center IɛD Okeanos, University of the Azores, Horta, Azores, Portugal; 16 Portuguese Institute for Sea and Atmosphere, Afonso Chaves Observatory, Ponta Delgada, São Miguel, Azores, Portugal; University of Namibia, NAMIBIA

## Abstract

Climate change can have direct and indirect effects on human health. Direct effects can include an increase in extreme weather events, such as heatwaves and floods, as well as an increase in the spread of vector-borne and infectious diseases, which may lead to a set of health problems and diseases. Indirect effects can include changes in air quality, water availability, and food production and distribution. These changes can lead to an increase in respiratory problems, malnutrition, and increased food insecurity. There is a perceived need to investigate the extent to which Higher Education Institutions (HEIs) are engaged in efforts to foster a greater understanding of the connections between climate change and health. In this context, this preliminary investigation offers an overview of the relationships between climate change and health. By means of a survey among teaching staff and researchers at HEIs from 42 countries across all continents working on the connection between climate change and health. The study has investigated the extent to which current provisions for education and training on the connection between climate change and health are being considered and how current needs in terms of policy development, research, and training are being met. A series of case studies illustrate how universities worldwide are actively developing strategies and implementing measures to address climate change and health. The study concludes by providing specific recommendations aimed at facilitating the handling of issues related to climate change and health in a higher education context.

## 1. Introduction

Climate change is one of the greatest threats to human health. It can impact human health in several direct and indirect ways, such as malnutrition, casualties, and physical injuries due to the intensified storms and floods that are termed as direct effects, droughts, water scarcity/contamination, heatwaves, increasing morbidity and mortality along with the adverse effects on mental health [1]. Indirect effects include an increase in tropospheric ozone, contributing to a rise in the number of asthma attacks and vector-borne diseases, such as malaria and dengue [2]. Risks for most health outcomes are projected to increase, with young children, the elderly, pregnant women, individuals with pre-existing morbidities, physical labourers, and people living in poverty, being the most vulnerable [3]. The increase in temperature and humidity in the atmosphere increases the body’s core temperature, resulting in a severe impact on heat illness and heat strokes. Extreme temperatures cause more heat-related deaths in addition to the illness of cardiovascular, kidney, and respiratory disorders [4].

An increase in thermal stress due to climate change can cause dehydration, tiredness, fainting, confusion, ineffective functioning of the brain, lack of concentration, and body discomfort [5, 6]. Environmental changes as a result of climate change are occurring swiftly. Rises in temperature contribute to the existence of more mosquitoes, ticks, and fleas, potentially resulting in an increased number of fatalities from diseases such as malaria. Mosquito types such as *Aedes*, *Anopheles*, and *Culex* transmit mosquito-borne infectious diseases [7, 8]. In addition, climate change is likely to increase the spread of vector-borne and infectious diseases such as dengue fever and Zika virus [9], as warmer temperatures and changes in precipitation patterns create more favourable conditions for disease-carrying mosquitoes and other vectors. According to [10], Semakula et al. [11], and Leedale et al. [12], malaria vector hotspots and prevalence in east and southern Africa and the Sahel are projected to increase under Representative Concentration Pathway (RCP) 4.5 and RCP8.5 by 2030 and becoming more pronounced later in the century [13].

Aside from the physical health impacts, climate change can also impact people’s mental health. As a direct result of an event related to climate change, individuals may immediately experience high stress, fear, terror, and being overwhelmed [14–16]. Following a climate change related event, individuals may experience a range of long-term mental health issues including displacement from home environments, post-traumatic stress, depression, grief and anger [14, 15]. Recent research by Hickman et al. [17] shows that the psychological burdens of climate change are affecting the mental health of young people across the globe.

Moreover, climate change can negatively impact the economy of regions and countries, which may subsequently lead to unemployment and socio-economic problems. Data indicates that in 2020, the world population experienced a loss of 295 billion potential work hours due to extreme heat exposure, with 79% of all losses occurring in countries with a poor Human Development Index, happening in the agricultural sector [18]. Considering the complex and wide-ranging impacts of climate change on human health, it is important to understand the relationships between climate change and health and to develop effective strategies to address these impacts.

There is a perceived need to foster knowledge of climate change and health as a whole, and of the contribution of Higher Education Institutions (HEIs) to this process, in particular. This significance arises from the unique position of universities as hubs of knowledge, research, and diverse expertise. Through interdisciplinary initiatives and research endeavours, universities play a pivotal role in catalysing innovative solutions to complex societal problems, particularly those associated with the profound challenges posed by climate change. Universities can provide research and resources to develop new technologies, promote public awareness of climate change, and offer educational opportunities to create a global network of knowledgeable citizens [19]. Universities can also influence policy decisions and create a platform for discussing the impacts of climate change. With the intensification of extreme weather events, the need to find ways to mitigate climate change is increasingly urgent. Accordingly, university engagement is essential to finding solutions to the environmental problems of today [19, 20].

There are several examples of activities that HEIs are already doing to address the health impacts of climate change and promote sustainability, namely:

- Education and awareness-raising. Many HEIs offer courses and programmes focused on sustainability and climate change [21–23]. For example, the University of California, Berkeley, offers a course in Global Public Health and Environment, which includes content on climate change and health [24]. The University of Cambridge in the UK offers a course on Climate Change and Health, which explores the impacts of climate change on public health [25].
- Research. HEIs are also leading research on the linkages between climate change and health [26, 27]. For example, Harvard University has established the Harvard Global Health Institute, which conducts research on the health impacts of climate change and other global health issues [28]. The Hamburg University of Applied Sciences in Germany hosts the Research and Transfer Centre "Sustainable Development and Climate Change Management", which among other efforts coordinates numerous university initiatives on climate change research, and also leads the world´s leading peer-reviewed book series on climate change, the "Climate Change Management" series: https://www.springer.com/series/8740 [29].
- Sustainable operations. Many HEIs are also implementing strategies to reduce their own carbon footprint [30]. For example, the University of Copenhagen, Denmark, has established a Climate Plan with the goal of reducing its carbon footprint and promoting sustainable practices. This includes initiatives such as energy-efficient buildings, green transportation, and sustainable food options.
- Community engagement. HEIs are also engaging with local communities to promote awareness of the health impacts of climate change and build partnerships to address these challenges [31]. For example, the National University of Singapore has established a Sustainability Office that focuses on promoting sustainability initiatives on campus and engaging with the wider community. This includes initiatives such as a Sustainable Campus program and a research group on climate change and health [32].

The cited examples represent only a handful of the numerous endeavours undertaken by HEIs worldwide to confront the health consequences of climate change and advance sustainability. While encouraging progress is observed in these collective initiatives, it is essential to recognize the existence of several obstacles that impede comprehensive engagement by universities in climate change-related efforts. Some of these are:

- Lack of awareness. Staff at certain universities may not possess a comprehensive awareness of the full extent of the impacts of climate change, including the significance of their role in actively engaging with this crucial topic [33].
- Limited resources. Several universities may lack the financial or human resources required to address the issue of climate change adequately [19].
- Unclear goals. Without well-defined goals, universities may find it challenging to prioritize their efforts and determine which strategies and initiatives are most effective in addressing climate change [34].
- Costs. Some initiatives for mitigating and adapting to climate change involve costs, and there could be political pressure from diverse stakeholders to avoid undertaking them [22].
- Short-term focus. Universities may prioritize short-term objectives, such as boosting enrolment or enhancing rankings, over long-term goals, such as reducing GHG emissions [19];
- Lack of continuity. It is noted that certain initiatives at universities have a short-lived duration, whereas a long-term perspective is essential [19].

In the broader context, universities are increasingly offering substantial support for various climate-related activities initiated and led by both their staff and students. This multifaceted engagement underscores a collective effort within the academic sphere to not only acknowledge the gravity of climate change but to actively contribute to sustainable practices, advocate for climate resilience, and foster a culture of environmental responsibility [35].

This preliminary study aimed to investigate these relationships and examine the extent to which current provisions for education and training on the connection between climate change and health are being considered and met in the context of HEIs. A research question was posed, specifically: How are HEIs addressing climate change and health issues in an integrated manner?

To answer this question, a mixed method approach was used. It combined a review of recent literature on the role of HEIs in addressing the connection between climate change and health, an international survey, and a set of selected case studies. The results of this research provide some useful lessons that may be applied by researchers, policymakers, and managers and staff in HEIs for improving their educational and research outcomes, as well as their social outcomes.

The article is structured as follows. First, the relationship between HEIs and climate change is explored from a comprehensive perspective. The subsequent section details the methods used in this research and provides information about the survey and the criteria used for identifying the case studies. This is followed by a thorough explanation of the results, which are cross-checked against the literature. The final section presents the main conclusions of this research and provides some recommendations for enhancing the role of HEIs in addressing the multiple relationships between climate change and human health.

## 2. Methodology

This preliminary investigation aimed to understand how HEIs are addressing climate change and health issues in a comprehensive manner. To answer this research question and ascertain the degree to which existing provisions for education and training on the nexus between climate change and health have been explored, along with identifying needs for policy development, research, and training, several methods have been applied. First, a survey has been conducted. The target population was teaching staff and researchers of HEIs working on the connection between climate change and health. In addition to this, a set of case studies were identified by the research team to better illustrate the areas in which universities are dealing with the connection between climate change and global health from different perspectives.

The questionnaire was developed by the team of authors following two main key areas of interest in the scope of this investigation:

**Education and training on Climate Change and Health.** This section was created with the aim of comprehending the respondents’ perception of the existing provisions on the topic. Questions focused on the following: i. provisions being sufficient to lead to lasting behavioural changes; ii. Interest of students/researchers on the topic of climate change and health; ii. interest of teaching staff/researchers on training on the topic of climate change and health; iii. level of satisfaction with training provisions (generally and at BSc, Master’s and Doctorate programmes); iv. main sources of information used for teaching/researching on climate change and health; v. aspects covered by teaching/research; and vi. aspects that demand further training. These aspects were based on Kotcher et al. [36] guidelines.
**Policy, research and training needs.** This section focused on the perception of respondents based on national contexts on the following: i. current initiatives on climate change and health; ii. barriers to the implementation of initiatives; iii. adequate availability of information regarding the impacts of climate on human health; iv. satisfaction on the emphasis given to provisions for climate change and health in the context of government policies; v. open space to share examples of specific policies on climate change impacts and health; vi. open space of final remarks.

In addition, the developed questionnaire also included an initial section dedicated to collecting demographic information for sample description (e.g., country, gender, age group, level of education, expertise and role in the higher education institution). The questionnaire is available in S1 Appendix.

After the pre-test, a stage in which an expert group of climate change and health researchers assessed the questionnaire and provided feedback that allowed the authors to reach a final improved revised version [37, 38]. The survey was then disseminated via the online tool Google Forms. Teaching staff and researchers associated with the International Climate Change Information and Research Programme (ICCIRP) were invited to respond to the survey, as well as those registered in academic networks and mailing lists related to climate change and health.

The nature of the research, the methods used, and the fact that no personal data was stored or can be traced back to individuals, conforming with General Data Protection Regulation (GDPR) standards, means that the study is not subject to an ethics permit, as specified by the Association of Medical Ethics Committee in Germany, the body responsible for such assessments in the country leading this study. In any case, and considering any argument requesting waiving consent, all respondents willingly agreed to participate in the study, confirmed through an additional question added to the beginning of the questionnaire, presenting options for yes or no. The survey collected responses during the second semester of 2022, from 5^th^ July to 28^th^ November and the results were analysed using descriptive statistics.

The survey was able to collect answers from 120 participants from 42 countries across all continents: Argentina, Austria, Bangladesh, Bhutan, Bolivia, Bosnia and Herzegovina, Brazil, Cameroon, Canada, Eritrea, Ethiopia, Gabon, Gambia, Germany, Ghana, India, Indonesia, Italy, Japan, Kenya, Kyrgyz Republic, Liberia, Malawi, Mexico, Mozambique, New Zealand, Niger, Nigeria, Pakistan, Portugal, Senegal, Serbia, South Africa, Spain, Sudan, The Netherlands, Tunisia, Uganda, United Kingdom, United States of America, Zambia, Zimbabwe. Table 1 presents the countries which were captured by this survey. It is possible to observe that 60% represent developing countries.

**Table 1:** Frequency of countries by alphabetical order (*n* = 42).

Country	Frequency of responses (%)
Argentina*	0.8
Austria	2.5
Bangladesh*	0.8
Bhutan*	0.8
Bolivia*	0.8
Bosnia and Herzegovina	0.8
Brazil	3.3
Cameroon*	1.7
Canada	1.7
Eritrea*	0.8
Ethiopia*	4.2
Gabon*	0.8
Gambia *	0.8
Germany	10.8
Ghana*	0.8
India*	9.2
Indonesia*	0.8
Italy	0.8
Japan	0.8
Kenya*	4.2
Kyrgyz Republic*	0.8
Liberia*	0.8
Malawi *	0.8
Mexico	0.8
Mozambique*	2.5
New Zealand	2.5
Niger*	0.8
Nigeria*	11.7
Pakistan*	1.7
Portugal	5.8
Senegal*	1.7
Serbia	0.8
South Africa*	2.5
Spain	1.7
Sudan*	3.3
The Netherlands	0.8
Tunisia *	0.8
Uganda*	4.2
United Kingdom	2.5
United States of America	4.2
Zambia*	0.8
Zimbabwe*	0.8

* Developing countries (60%).

**Fig 1:**
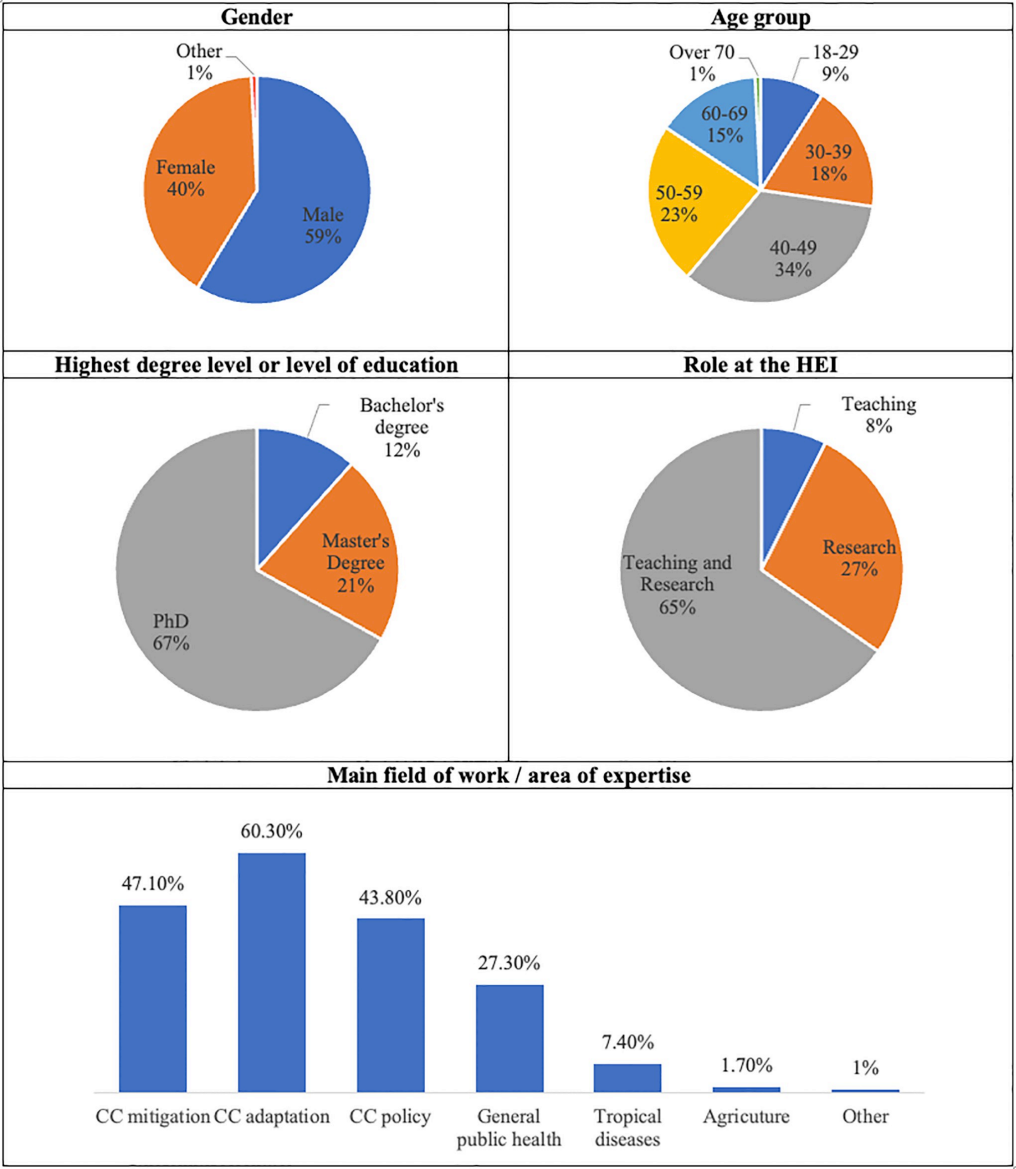
Demographic and professional characteristics of participants (%).

The first section of the survey was used to collect demographic and professional information about participants in this research. There are no significant differences in terms of balance among the surveyed developed and developing countries, relating gender, age, degree level and role at the HEI. In terms of field of work or expertise area, CC mitigation is more present in developing countries (near 3/4 versus ¼), CC adaptation is more present in developing countries, as well and CC policy (around 2/3 versus 1/3) and general public health is more present in developed countries (2/3 versus 1/3), an expected characterization, explained by the dominant issues. Most relevant information is shown in Fig 1.

In addition to this, the results are complemented by a set of cases studies which present how universities across the globe are working to formulate strategies and take measures aimed at mitigating the impact of climate change on health. Adopting Yin’s [39] classification, the cases were classified as type I. Each case was examined holistically to showcase the strategies implemented. This comprehensive approach facilitated the study’s explanatory power. The analysis of the case studies has been carried out by showcasing the approach used to foster awareness on the connections between climate change and health, and the results obtained. The latter is meant to serve as inspiration to other organisations willing to, but reluctant to engage in this field.

## 3. Results and discussion

### 3.1 Survey

**Fig 2:**
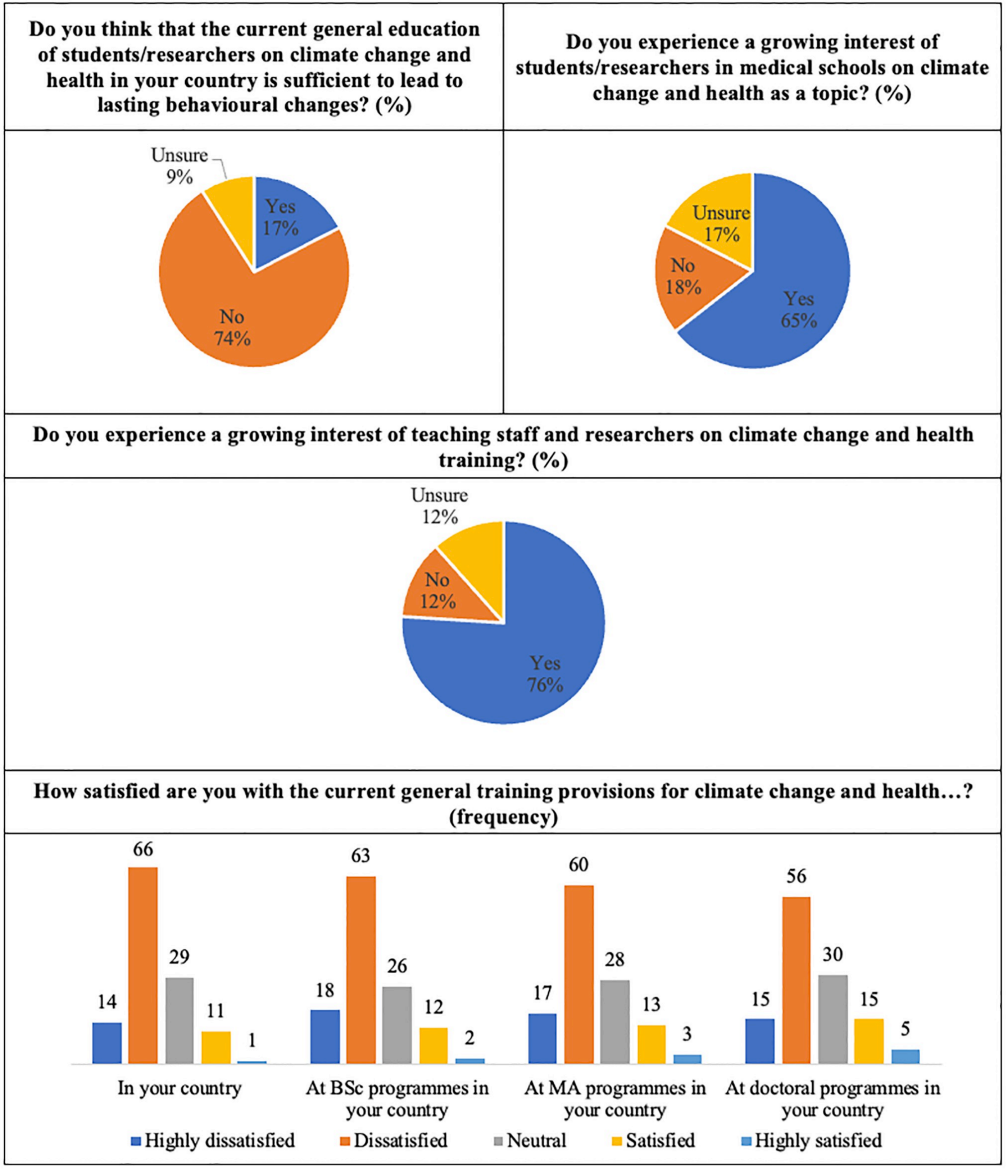
Participant’s perception regarding the efforts carried out in their HEIs.

After section 1, focused on demographics, described above, section 2 of the enquiry aimed to assess *the current provisions for education and training on climate change and health in their countries*. The first 4 questions in this section aimed to assess the participant’s perception regarding the efforts carried out in their HEIs. The results, which are in line with common concerns raised by researchers regarding the need for increased efforts in HEIs to stress the climate emergency [40], are shown in Fig 2.

In this regard, 74.2% of the respondents acknowledge that *current general education of students/researchers on climate change and health in their country* is **not** *sufficient to lead to lasting behavioural changes*, while 16.7%, mostly located in developing countries (e.g., Bhutan, Ethiopia, India, Nigeria, Pakistan, Senegal Sudan or Uganda), say it is. As regards the interest for this topic in medical schools, 64.2% of the respondents have reported to *experience a growing interest of students/researchers on climate change and health as a topic*. This value is higher (75.8%), when answering the question whether *experiencing a growing interest of teaching staff and researchers on climate change and health training*, with a negative answer being given by the same percentage of developing and developed countries.

Participants were also questioned about their *level of satisfaction regarding the current training provisions for climate change and health in their countries*, taking into consideration the different educational and training levels. As regards the general context, 55.0% of the respondents reported being dissatisfied, 24.2% remained neutral, 11.7% were highly dissatisfied and 9.2% were satisfied. In relation t*o the level of satisfaction with the current training provisions for climate change and health at* ***BSc*** *programmes*, 52.5% reported dissatisfaction, followed by neutrality in 21.7%, 15.0% being highly dissatisfied, 10.0% being satisfied and 0.8% being highly satisfied. These values are similar when respondents are now questioned about the same topic for ***Maste****r’s programmes*, were 50.0% are dissatisfied, 23.3% are neutral, 14.2% are highly dissatisfied, 10.8% are satisfied and 1.7% are highly satisfied, with the results to both questions showing a close connection between the BSc and Master programmes engagement. Considering the ***PhD*** *programmes*, the answers are also close to the set of responses reported to the BSc and Master programmes. The responses obtained to the same question for this educational level were, per order, the following: 46.7% dissatisfied, 25.0% neutral, 12.5% highly dissatisfied, 12.5% satisfied, and 3.3% highly satisfied, revealing again, how this cycle of studies gets near the results obtained to the other cycles. and they are aligned with previous research showing concerns regarding the fact that educational actions need to be reinforced to increase awareness and comprehension among students [41]. It is possible to observe that the dissatisfaction level decreases with the level of education increase, from the BSc, master, to PhD, which makes sense, considering that higher education levels will correspond to a higher level of engagement and environmental awareness in this respect. In any case, the percentage corresponding to a high degree of satisfaction is extremely low, which highlights the need for further investment in training provisions involving climate change and health cross-cutting issues. These relative levels of dissatisfaction are aligned with concerns regarding the relatively low levels of investment to foster interdisciplinary research [42], which is a core component of research-based education in all educational levels and training approaches, either in developing or developed countries.

The respondents were also enquired about the *main sources of information used for teaching/researching on climate change and health*. The answers illustrate the diversity of opinions collected, from books, international reports/guides, IPCC reports, or scientific articles.

Enquired about the *aspects already covered in the teaching and/or research* developed by the respondents, the answers proved a wide scope of interests.

**Fig 3:**
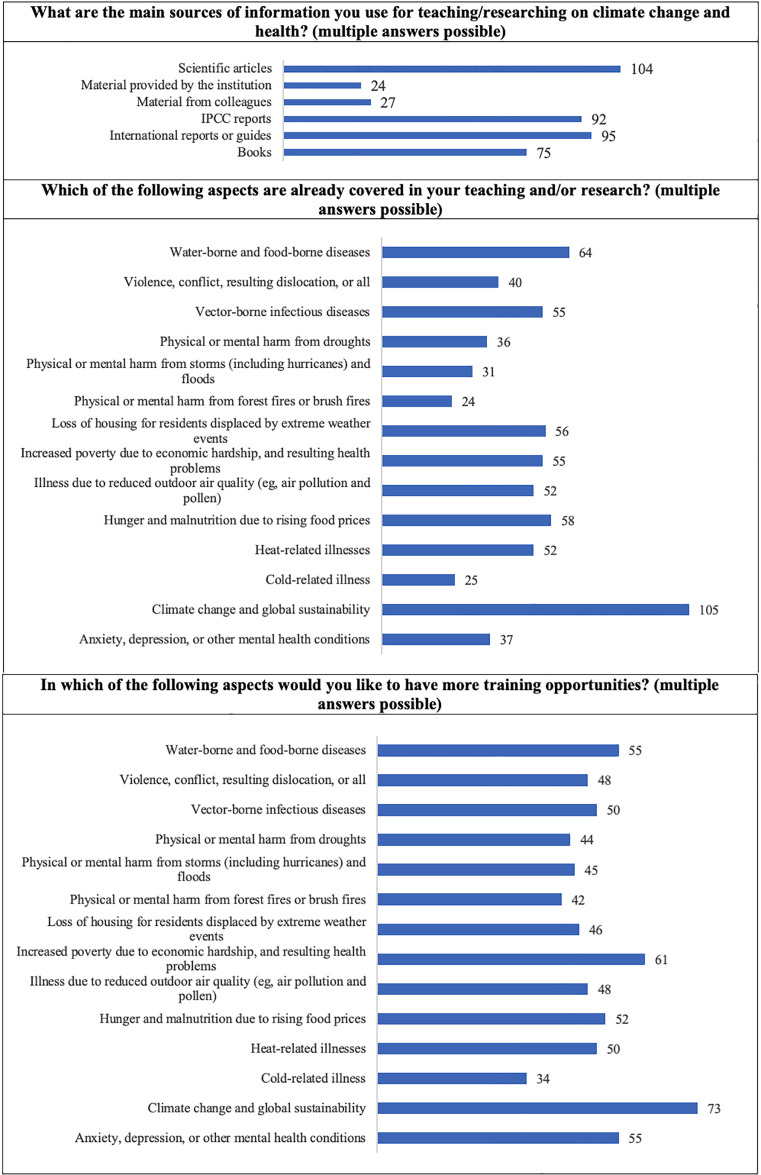
Sources of information, areas covered in research, and willingness to receive training (frequencies).

Involving the same set of answers’ options and enquired about *which of the aspects would the respondents like to have more training opportunities*, the responses obtained were mostly distributed evenly. These answers show how eager the respondents are about acquiring knowledge that involves all issues related to climate change one way or the other, while, at the same time the climate change and global sustainability option of answer was selected representing an equal set of respondents percentage, for whom the chosen terminology represents it all there is to know about climate change, thus revealing a gap in knowledge at this respect, that need to be further addressed, as suggested by Leal Filho et al. [22, 23]. These results are detailed in Fig 3.

**Fig 4:**
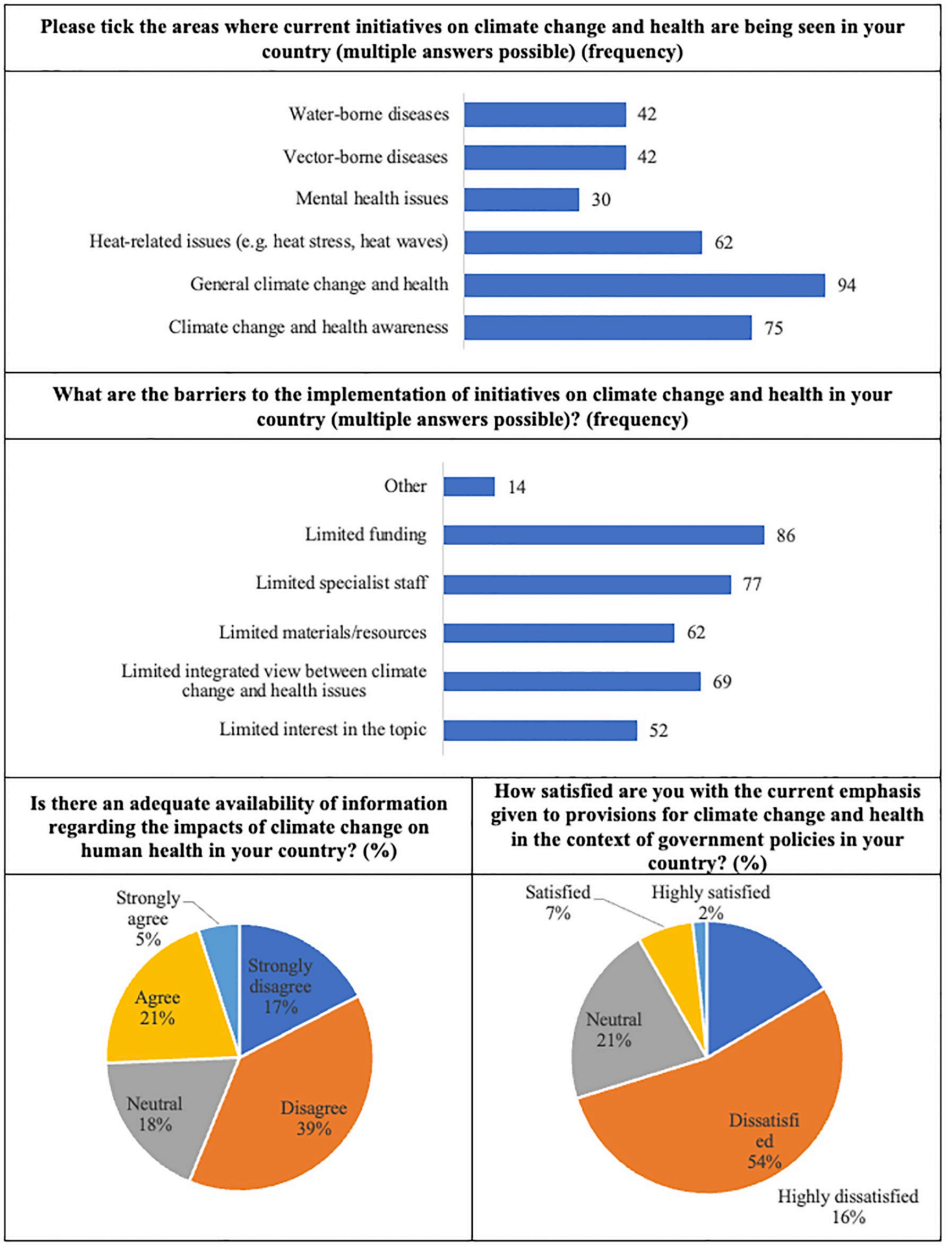
Participant’s opinion regarding several initiatives or actions in their countries.

The third and last section of the survey is focused on *policy*, *research and training needs*. It included four questions aimed to assess the *areas where current initiatives on climate change and health are being seen in the respondents’ country*, the *barriers to the implementation of initiatives on climate change and health in the respondents’ country*, and the participant’s opinion regarding *the impacts of climate change on human health in the respondents’ country*. The answers to these questions are shown in Fig 4. These results are consistent with previous research, suggesting that relevant social groups may not be sufficiently targeted when designing climate change and health related policies, or information strategies and campaigns, and contribute to better understand the reasons for these concerns [43–45].

Finally, to the question enquiring whether there are *examples of specific policies on climate change impacts and health to be mentioned by the respondents*, many of the respondents opt not to answer. However, 44.16% of participants have given specific examples, highlighting issues that should be improved in areas like implementation, policies and regulation, quantity and quality of information, education and training, and awareness/understanding or need of research. These findings illustrate the main areas for concern among citizens [46, 47]. At this respect, Table 2 includes statements from the participants aiming to illustrate specific contexts, within different countries, mostly from developing nations, in these cases, but not only.

**Table 2:** Participants examples of specific policies on climate change impacts and health.

Context Analysis	Participant statement
Most of the answers related to implementation issues.	*“The truth is that policies on climate change impacts and health documents exist*. *However*, *the level of implementation is alarmingly low*, *which puts the whole system in danger of failure*, *thus increasing the communities’ vulnerability and risk*.*” (Participant 1*, *Uganda)*
Several participants stressed the need for improvement in quality and quantity of information regarding climate change and health, and the potential challenges that it may entail.	*“Climate change impacts on health are commonplace*, *as they happen everywhere*. *However*, *the documentation of this phenomenon in developing countries is still scanty if it exists*. *For instance*, *it is difficult to establish whether a certain number of admissions to health facilities is due to climate-induced diseases*. *This is a serious gap that needs research to fill the knowledge gap*.*” (Participant 2*, *Uganda)*
Several respondents comment on the necessary improvements that are still required in higher education to enhance the role that universities and other institutions must play in becoming drivers for real change	*“An attention should be given for mainstreaming of climate change impacts and health in our curriculums and research and community service activities*. *They put policies in place but never implement it*. *For example*, *3rd year students don’t even know what blue carbon is because they do not have background knowledge of the subject*.*” (Participant 3*, *Ethiopia)*
Some respondents identified specific issues that make it difficult for the people to fully understand the effects of climate change on health, therefore highlighting the need for more research on the topic and better dissemination among different audiences.	*“Climate change and health is a multidimensional and systemic topic—very difficult to identify a single issue*. *At the same time*, *it is necessary to take into consideration vulnerable persons*, *women and the youth” (Participant 4*, *United Kingdom)*

A final query in section three allowed the respondents to leave final remarks. 39.0% or participants leave some interesting comments regarding issues like education, awareness and understanding, stakeholder collaboration, regional or global networks. Table 3 illustrates the associated challenges reported.

**Table 3:** Participants examples of specific challenges on climate change impacts and education.

Implications	Participant statement
Regarding education, several participants expressed their concerns regarding the relatively low level that climate change and health issues have in some countries.	*“I think raising awareness has to start earlier*, *ideally at school*, *and not only on climate change as such (it is getting warmer)*, *but also on the health aspects*.*” (Participant 5; Austria)*
Literacy is particularly relevant for developing countries, where these challenges may be causing larger effects, especially due to the lower resources that may be available for adaptation and resilience strategies	*“At community level*, *in order to address the extensive damage that humans have created and acknowledge our health is intrinsically linked to the health of the planet*, *it is important to integrate such discussions with health literacy*. *This planetary health and health literacy integration has been championed by the West African Institute of Public Health and advocated to institutions that run public health programs in West Africa*.*” (Participant 6*, *Nigeria)*
In line with this need of enhanced education, several participants recognized that there is much room for improvement regarding public awareness and understanding.	*“It’s sometimes difficult to link some health issues to climate change impacts*. *Particularly among rural households who could not relate their experiences with the climatic variations affecting their health and agricultural productivity*.*” (Participant 7*, *Nigeria)*
Another issue addressed by several respondents refers to the role that regional and international networks play in fostering collaboration among different stakeholders, both from the public and private sectors, and between the global South and the North.	*“Is necessary to enhance the international network of climate change and health*. *In south hemisphere we saw first climate change and health impacts*, *considering the poverty and pre-existent vulnerabilities*.*” (Participant 8*, *Bolivia)*

Finally, it is interesting to note that several participants expressed their interest in increasing their involvement in this area of research, and to contribute to the creation or reinforcement of regional or global networks focused on this topic [40, 48].

To sum up, the results obtained in the survey involving respondents from across various geographical regions do demonstrate the need to further invest in climate change and health related education. HEIs have an essential role in dissemination of information and act as drivers of change within the society. Accordingly, and as highlighted in the results obtained, it becomes imperative to further invest so that the studied cycles of study are able to engage students, teachers and researchers to further commit with these issues, that will contribute to promote an entire societal change, through the increased environmental awareness towards climate change and health impacts.

### 3.2 Case studies: Universities as key players in addressing climate change and health

Around the world, there are already several inspiring projects from leading universities in climate change and health. Table 4 illustrates how universities across the globe are working to formulate strategies and take measures aimed at mitigating the impact of climate change on health. This collecting of information serves the purpose of contextualizing the data obtained from the survey. This process enhances the overall understanding and interpretation of the collected data by providing additional background and context, ensuring a more comprehensive analysis of the specific role played by HEIs in climate change and health initiatives. Naturally, the examples presented in the table mostly focus on developed countries, more easily found in the scientific literature. This focus might arise due to a higher prevalence of research activities, resources, and data accessibility in developed nations. However, despite this imbalance, the examples laid out in the table are intended to serve as recommendations for actions to be pursued by developing countries. By examining the experiences and strategies implemented by developed countries, policymakers, researchers, and practitioners in developing countries can derive valuable guidance. Additionally, this approach encourages a collaborative and adaptive approach to problem-solving. Developing countries can adapt and tailor successful strategies to their unique circumstances, considering factors such as cultural differences, resource availability, and healthcare infrastructure, emphasising the importance of shared learning and leveraging global experiences to accelerate progress in addressing common challenges faced by both developed and developing nations.

**Table 4:** Examples of inspiring projects from universities worldwide addressing climate change and health.

Institution	Case study title	Approach used	Impact	Reference
Hamburg University of Applied Sciences, Germany	Research and Technology Transfer Sustainability and Climate Change Management	Training on climate change Editing book series on climate change	Over 3.000 academic staff Over 15 books Participation at IPCC´s Assessment Reports	[22, 49, 50]
University of the Witwatersrand, Johannesburg, South Africa	Teaching and Learning for Climate Change	Teacher education materials development, high school material design, and the design and implementation of a new tertiary-degree offering centred on teaching and learning for climate change	University-level graduates, teachers, facilitators of learning, young school-going learners and adult learners	[51]
School of Public Health & Family Medicine, University of Agriculture and Natural Resources, Malawi	Training needs assessment for climate change and in relation to health	Cross sectional qualitative study design involving primary data collection and review of curricula documents of undergraduate and postgraduate programmes offered at several HEIs	1 book; Reviewed curricula for several Bachelor and Master degree; Trainings in gender, climate change and intersection with health	[52]
International Federation of Medical Students Associations (IFMSA), Denmark	Research about planetary health in every medical curriculum	Student exchange program; Research project	Planetary health in the medical curriculum; More than 15,000 students per year, more than 130 countries involved; Practice medicine or medical research in a new cultural environment	[53, 54]
Yale School of Public Health, USA	Climate Change and Health	Online Certificate Program	Build a professional network, forge connections with other participants	[55]
University of Miami, USA	Varied strategies	Master program of Science in Climate and Health, research, various thesis	Train students in understanding, evaluating, and assessing short- and long-term climate and weather changes, their direct and indirect impact on disease and disability burden; Prepare students to develop adaptation, mitigation, healthcare and communication strategies	[56]
American Association of Colleges of Nursing, USA	AACN’s programs	Webinars	Training for nursing educators who teach in bachelor’s and graduate programs	[57]
University of Sydnei, Australia	Human Health and Social Impacts Node Research	Forum	Sources of health and climate change information	[58]
Australian National University, Australia	College of Health & Medicine	Research projects	Health policy	[59]
University of Nairobi	Institute for Climate Change and Adaptation (ICCA)	Plenary session	Transformative practices that can help better understand planetary health and its implications for the African continent	[60]
Columbia University, USA	Global Consortium on Climate and Health Education	Work together with health professionals to prevent, reduce, and respond to the health impacts of climate change. Courses	300 health professional member institutions from 56 countries; Reaching an estimated 175,000 students annually More than 240 member institutions and partners	[61]
University of Colorado, USA	The consortium takes lead in studying climate change effects on health. Climate & Health Program	Research, education	Research and community partnerships set up, faculty members engage with health care professionals	[62]
University of Washington, USA	CHanGE	Mentoring, certificate program, technical review, climate change modeling	Prioritize health in climate change, mitigation, and adaptation, incorporate climate resilience into all health sector activities	[63]
Heidelberg University Hospital, German	Massive Open Online Course—MOOC	E-learning formats on climate change and health for higher education, health staff, and policy makers	23 000 students passed; 253 got perfect scores; 410 online students outperformed the top	[64, 65]
Johns Hopkins Bloomberg School of Public Health, USA	Climate and Health Certificate Program	Courses explore the effects of energy production and climate change on food, water, air, and human health, through the lens of social justice.	Prioritize climate change and its effects on public health, and ways to mitigate the impacts	[66]
University of Amsterdam, Netherlands	Teaching in climate change and planetary health	Planetary health module in a medical curriculum	More than 1,700 researchers from multidisciplinary disciplines; Integration of planetary health and climate change into the competencies of future health workers	[67, 68]
University of Tokyo, Japan	Integrated Research System for Sustainability Science of the University of Tokyo (IR3S)	International academic journal; Editing academic books in sustainability science;	17 series academic Journal 3 academic books	[69, 70]
Barcelona Institute of Global Health, Spain	UOC-UPF-ISGlobal Program	Master of Planetary Health	Add skills to workers at NGOs, sanitary services, and private business sector	[71]
University of São Paulo, Brazil	Global Health and Sustainability Program (PPG-SGS)	PhD in consolidating the field of critical studies on global health, exploring its interface with sustainability	Provide skills to researchers, public agents, and other professionals working in health and the environment	[72]
University of Ghana	Teaching and Learning for Public Health-Undergraduate Program	Bachelor of Public Health (BPH)	Professionals with mid-level technical and leadership skills for the health sector in Ghana	[73]

The topics covered in Table 4 are diverse but all related to sustainability, health, and well-being. Overall, these topics illustrate the importance of understanding climate change in the context of health, sustainability, and well-being in various settings. They also highlight some educational interventions research, and collaboration aimed at addressing these critical issues.

One area where action is also needed is in respect of training needs assessment for climate change and health. These can guide the preparation of teacher education materials, new degree programmes, and the integration of matters related to climate change and planetary health into the curriculum of health professions, training students to understand, evaluate, and assess climate changes and their impacts on health, fostering the development of adaptation and mitigation strategies. Thus, contributing to health policy or focusing on transformative practices for understanding planetary health in the context of the African continent are only some of the undertakings summarized above.

Universities can provide a key contribution to global efforts to fight against climate change. Their ability to generate knowledge, promote collaboration, and develop innovative solutions, by means of research, public awareness, education, and policy-making is more critical than ever [19, 20, 22], especially if one takes into account the increasing urgency to address climate change.

## 4. Conclusions

This paper has reported on a study on the current state of education and training on climate change and health across 42 countries worldwide, i.e., Argentina, Austria, Bangladesh, Bhutan, Bolivia, Bosnia and Herzegovina, Brazil, Cameroon, Canada, Eritrea, Ethiopia, Gabon, Gambia, Germany, Ghana, India, Indonesia, Italy, Japan, Kenya, Kyrgyz Republic, Liberia, Malawi, Mexico, Mozambique, New Zealand, Niger, Nigeria, Pakistan, Portugal, Senegal, Serbia, South Africa, Spain, Sudan, The Netherlands, Tunisia, Uganda, United Kingdom, United States of America, Zambia, Zimbabwe. The survey was conducted among teaching staff and researchers at HEIs working in this field, aimed at identifying gaps in knowledge and skills among educators and researchers, as well as the challenges and opportunities associated with integrating climate change and health into the higher education curricula. The findings from the survey including participants from diverse geographical regions, unequivocally underscore the urgency of increased investments in education about climate change and its intersection with health. HEIs are pivotal entities in the dissemination of crucial information and promotion of training on climate change. As such, they are also catalysts for societal transformation. As delineated by the survey results, it is important to intensify efforts at universities, with educational programs to effectively involve both, staff and students, as well as the organisations they work with. This heightened commitment is indispensable for fostering a comprehensive societal shift, marked by heightened environmental consciousness regarding the implications of climate change on health.

By fostering awareness and promoting engagement at various educational levels, HEIs can collectively contribute to ushering in a broader societal change in attitudes and actions towards these pressing issues.

The information collected through case studies shows that various initiatives have been undertaken in HEIs, including conducting training needs assessments for climate change and health, creating teacher education materials, or training students to comprehend and assess climate change impacts on health. These endeavors also involve fostering the development of adaptation and mitigation strategies, contributing to health policy, and focusing on transformative practices for understanding planetary health. Together, these initiatives reflect a global commitment within HEIs to address the intersection of climate change and health, showcasing diverse approaches and impacts.

This study has some limitations. The first one is the fact that the empirical part was undertaken over a short period of time. A further limitation is related to the fact that the sample, with 120 participants, was not large enough to allow a more robust statistical approach to be used. Nonetheless, this study provides a welcome contribution to the literature since it has analysed and documented trends related to the ways in which HEIs in 42 countries currently handle provisions related to climate change and health, which makes this study one of the most comprehensive ones on the topic, while limited to the sample. It is challenging to collect information on the topic among so many countries.

The geographical distribution of the sample, including 42 countries from all the continents, offers a rough profile of international trends, hence helping to foster a broader understanding of the international implications of this important topic. Future studies may aim to collect a wider range of responses, thus catering to more complex statistical analyses.

To effectively tackle the challenges pinpointed in the study and foster the seamless integration of climate change and health into higher education curricula, several recommendations can be proposed. For instance:

- There should be a heightened emphasis on developing interdisciplinary programmes and courses that seamlessly integrate the intricate dimensions of climate change and health. This approach ensures a comprehensive educational experience that transcends disciplinary boundaries.
- To enhance the educational landscape, there is a need to integrate climate change and health topics into existing courses, especially those in environmental science, public health, and biology. This incorporation enriches the curriculum by infusing relevant content into established academic domains.
- Pioneering the creation of new interdisciplinary courses dedicated to the exploration of climate change and health is imperative. These courses should draw upon insights from various disciplines, fostering a holistic understanding of the multifaceted relationship between climate change and health.
- Establishing experiential learning opportunities, such as field trips and research projects, is crucial for a hands-on exploration of climate change and health topics. These opportunities provide students with real-world exposure and deepen their understanding through practical engagement.
- To further enrich the educational experience, it is essential to develop avenues for students to directly engage with experts and practitioners in the field. This interaction facilitates a direct exchange of knowledge, insights, and experiences, enhancing students’ comprehension of the complex issues at the intersection of climate change and health.
- Facilitating meaningful conversations among students, faculty, and practitioners about the interconnectedness of climate change and health is vital. Creating spaces for dialogue fosters a collaborative learning environment, encouraging the exchange of perspectives and the development of well-rounded insights into the subject matter.

By implementing these recommendations, universities can not only address the identified challenges but also contribute significantly to preparing future professionals with a comprehensive understanding of the critical nexus between climate change and health.

Finally, greater provisions should be made for training, so that teaching staff and researchers may become more familiar with the connections between climate change and health and, by doing so, feel encouraged to handle these issues in their study programmes and research. In this context, the establishment of partnerships between HEIs and public health organisations may prove fruitful, promoting collaboration and knowledge exchange on matters related to climate change and health, to the advantage of current and future university students. This is supported by the fruitful cases studies presented, underscoring the transformative potential of using these examples as a guide for developing countries, thus promoting a cross-pollination of ideas and strategies, facilitating a more inclusive and collaborative approach to tackling shared issues on a global scale.

## Supporting information

S1 Appendix Study on climate change and health—Survey question. (DOCX)

S1 Dataset (XLSX)
