# Supplementary material for: Assessing climate change and health provisions among staff in higher education institutions: A preliminary investigation
Source: PLoS One. 2024 May 21;19(5):e0304019. doi: 10.1371/journal.pone.0304019 (PMC11108151; doi:10.1371/journal.pone.0304019)
Supplement: S1 Appendix — (DOCX) [file pone.0304019.s001.docx]

**S1 Appendix. Study on Climate Change and Health - Survey Questions**

**Section 1. Demographic information**

1. Your country.

2. Your gender.

3. Your age group.

4. Highest degree level or level of education.

5. Field of work/expertise. (checkboxes).

6. Your role at the HEI. (multiple choice possible).

**Section 2. Current provisions for education and training on Climate Change and Health.**

7. Do you think that the current general education of students/researchers on climate change and health in your country is sufficient to lead to lasting behavioural changes? (multiple choice possible).

8. Do you experience a growing interest of students/researchers in medical schools on climate change and health as a topic? (multiple choice possible).

9. Do you experience a growing interest of teaching staff and researchers on climate change and health training? (multiple choice possible).

10. How satisfied are you with... ? (multiple choice grid).

11. What are the main sources of information you use for teaching/researching on climate change and health? (checkboxes).

12. Which of the following aspects are already covered in your teaching and/or research? (multiple answers possible) (checkboxes).

13. In which of the following aspects would you like to have more training opportunities? (multiple answers possible) (checkboxes).

**Section 3. Policy, research and training needs.**

14. Please tick the areas where current initiatives on climate change and health are being seen in your country (multiple answers possible) (checkboxes).

15. What are the barriers to the implementation of initiatives on climate change and health in your country (multiple answers possible)? (checkboxes).

16. There is an adequate availability of information regarding the impacts of climate change on human health in your country. (multiple choice possible).

17. How satisfied are you with the current emphasis given to provisions for climate change and health in the context of government policies in your country? (multiple choice possible).

18. Are there examples of specific policies on climate change impacts and health you would like to mention? Please feel free to use this space.

19. Please let us know if you have any comments or wish to add/highlight anything.
